# The Effect of Cotrimoxazole Prophylactic Treatment on Malaria, Birth Outcomes, and Postpartum CD4 Count in HIV-Infected Women

**DOI:** 10.1155/2013/340702

**Published:** 2013-12-05

**Authors:** Anna Dow, Dumbani Kayira, Michael G. Hudgens, Annelies Van Rie, Caroline C. King, Sascha Ellington, Nelecy Chome, Athena Kourtis, Abigail Norris Turner, Zebrone Kacheche, Denise J. Jamieson, Charles Chasela, Charles van der Horst

**Affiliations:** ^1^University of North Carolina Gillings School of Global Public Health, 2104 McGavran-Greenberg Hall, 135 Dauer Drive, CB 7435, Chapel Hill, NC 27599-7435, USA; ^2^UNC Project-Malawi, Tidziwe Centre, Private Bag A-104, Lilongwe, Malawi; ^3^University of North Carolina Gillings School of Global Public Health, 3107-E McGavran-Greenberg Hall, Chapel Hill, NC 27599, USA; ^4^University of North Carolina Gillings School of Global Public Health, 2104 McGavran-Greenberg Hall, Chapel Hill, NC 27599-7435, USA; ^5^US Centers for Disease Control and Prevention, 1600 Clifton Road, Atlanta, GA 30333, USA; ^6^The Ohio State University, N1145 Doan Hall, 410 W. 10th Avenue Columbus, OH 43210, USA; ^7^Epidemiology and Biostatistics Division, School of Public Health, Faculty of Health Sciences, University of Witwatersrand, Johannesburg, South Africa; ^8^Division of Infectious Diseases, University of North Carolina, 130 Mason Farm Road, Chapel Hill, NC 27599-7030, USA

## Abstract

*Background*. Limited data exist on cotrimoxazole prophylactic treatment (CPT) in pregnant women, including protection against malaria versus standard intermittent preventive therapy with sulfadoxine-pyrimethamine (IPTp). *Methods*. Using observational data we examined the effect of CPT in HIV-infected pregnant women on malaria during pregnancy, low birth weight and preterm birth using proportional hazards, logistic, and log binomial regression, respectively. We used linear regression to assess effect of CPT on CD4 count. *Results*. Data from 468 CPT-exposed and 768 CPT-unexposed women were analyzed. CPT was associated with protection against malaria versus IPTp (hazard ratio: 0.35, 95% Confidence Interval (CI): 0.20, 0.60). After adjustment for time period this effect was not statistically significant (adjusted hazard ratio: 0.66, 95% CI: 0.28, 1.52). Among women receiving and not receiving CPT, rates of low birth weight (7.1% versus 7.6%) and preterm birth (23.5% versus 23.6%) were similar. CPT was associated with lower CD4 counts 24 weeks postpartum in women receiving (−77.6 cells/**μ**L, 95% CI: −125.2, −30.1) and not receiving antiretrovirals (−33.7 cells/**μ**L, 95% CI: −58.6, −8.8). *Conclusions*. Compared to IPTp, CPT provided comparable protection against malaria in HIV-infected pregnant women and against preterm birth or low birth weight. Possible implications of CPT-associated lower CD4 postpartum warrant further examination.

## 1. Introduction

Cotrimoxazole prophylaxis has been shown to reduce morbidity and mortality in HIV-infected adults and children [1–5]. The World Health Organization (WHO) guidelines issued in 2006 recommend daily cotrimoxazole prophylactic treatment (CPT) for HIV-infected adults and HIV-infected pregnant women with CD4 cell counts of less than 350 cells/*μ*L or WHO clinical stage III or IV [6]. The WHO guidelines advised adapting the CD4-based CPT eligibility cut points based on availability of CD4 testing and country-specific resources. Data on CPT in HIV-infected pregnant women are scarce, though there is some evidence suggesting that CPT may reduce the risk of poor birth outcomes in women with CD4 cell counts of less than 200 cells/*μ*L in addition to reducing morbidity and mortality due to opportunistic infections [7].

CPT in HIV-infected adults has been associated with reduced malaria incidence [1, 3, 8]. HIV-infected pregnant women may have greater benefit from malaria prophylaxis, as these women experience more peripheral and placental malaria compared with HIV-uninfected pregnant women [9]. Due to similarities between cotrimoxazole and sulfadoxine-pyrimethamine (SP), SP-based intermittent preventive therapy during pregnancy (SP-IPTp) for malaria, which is usually given to women during pregnancy regardless of HIV status, is not given in cases where CPT is given [6]. Thus far, only one cross-sectional study has assessed the effect of CPT on malaria among HIV-infected pregnant women [10].

In the present analyses, we examined the effect of CPT initiated during pregnancy in HIV-infected women with a CD4 cell count between 200 and 500 cells/*μ*L on adverse maternal and infant outcomes and on change in CD4 cell count from time of screening until 24 weeks postpartum. These analyses on CPT will add to the limited knowledge on this component of HIV care, which is widely used in highly vulnerable populations.

## 2. Methods

### 2.1. Study Design and Population

All women included in this analysis were enrolled in the Breastfeeding, Antiretrovirals, and Nutrition (BAN) randomized, controlled trial, which took place in Malawi between 2004 and 2009 [11]. BAN's study design and primary findings have been reported elsewhere [11, 12]. Briefly, ART-naïve, pregnant, HIV-infected women at least 14 years of age and ≤30 weeks gestation were eligible for enrollment if they had hemoglobin levels >7 g/dL, CD4 cell count ≥250 cells/*μ*L (≥200 cells/*μ*L before July 24, 2006), normal liver function tests (ALT <2.5 × the upper limit of normal), and no serious pregnancy complications. Depending on the estimated gestational age at screening, women were asked to return for follow-up prenatal care at approximately 28, 32, and 36 weeks' gestation.

For the BAN Study, if mother-infant pairs met secondary eligibility criteria, they were randomized within one week of birth to a two-group maternal nutritional intervention and a three-group antiretroviral intervention consisting of a triple-drug antiretroviral regimen for the mother (maternal-regimen group), daily dose of nevirapine for the infant (infant-regimen group), or neither (control antiretroviral group). The interventions began after delivery and were continued until the cessation of breastfeeding but no longer than 28 weeks. Infants found to be HIV infected at birth or in the first 2 weeks of life were disenrolled from the BAN study and referred for care. Study follow-up visits took place every 1–6 weeks, and data for the current analysis came from visits during pregnancy, delivery, and 24 weeks postpartum.

In accordance with the Malawi Ministry of Health and Population guidelines and WHO guidelines on cotrimoxazole prophylaxis [13], CPT was initiated in the BAN Study for eligible women and infants in 2006. Starting on 13 June 2006, CPT (480 mg twice daily) was provided after the 12th week of pregnancy to all participating women with a CD4 cell count of less than 500 cells/*μ*L, regardless of symptoms. CD4 cell counts were performed at screening and 24 weeks and 48 weeks postpartum. CPT could be started based on a CD4 cell count of less than 500 cells/*μ*L at any of those time points. The routine second and third trimester doses of SP given to pregnant women were omitted in women receiving CPT in accordance with WHO recommendations [6]. Once initiated, CPT was intended to be lifelong and was provided for the duration of study participation by the BAN Study. Women who enrolled before CPT was initiated were provided with SP-IPTp as per Malawi's national guidelines which was administered as two 1575 mg doses of SP; one dose during the second trimester and one dose during the third trimester. Specific data on coverage within the study population was not available. In 2010, the estimated coverage for both doses of SP during pregnancy in Malawi was approximately 55% [14], though higher rates have been reported within Malawi [15].

The BAN Study's protocol was approved by the Malawi National Health Science Research Committee and the institutional review boards at the University of North Carolina at Chapel Hill and the US Centers for Disease Control and Prevention.

### 2.2. Definitions

**Figure 1:**
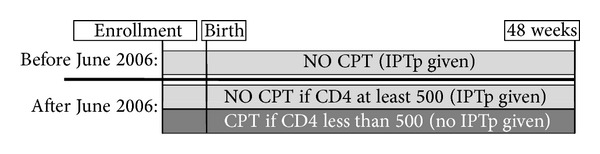
In the BAN Study, women were enrolled during pregnancy and followed until 48 weeks postpartum. Prior to June 2006, all women received SP-IPTp as recommended by the Malawi Department of Health. Following WHO recommendations, after June 2006 CPT was implemented for women with a CD4 of less than 500 and SP-IPTp was discontinued for these women. Women with a CD4 of 500 or greater continued to receive SP-IPTp.

The unanticipated change in CPT guidelines and their implementation 2 years into the BAN Study created a natural experiment, with a CPT-unexposed period followed by a CPT-exposed period (Figure 1). Therefore, for the purpose of this analysis, exposure to CPT was based on the 2006 time point at which standardized CPT was implemented in the BAN Study. To minimize misclassification of CPT, inclusion in our analysis was restricted according to 2 criteria. First, in order to account for any lag time between the decision to administer CPT and the routine implementation of this practice, women presenting for their second prenatal visit (median time of 12.7 weeks before delivery) between 13 June 2006 and 15 August 2006 were not included in these analyses. Second, analyses only included women who were either never exposed to CPT or women who were exposed from their second prenatal visit onwards.

Malaria was defined as the first episode after the second prenatal visit and was diagnosed by a positive blood smear from a woman presenting with malaria symptoms (including fever >38 degrees C, sweats, chills, malaise, headache, or pallor). Because of the short follow-up period, women with a documented diagnosis of malaria at or before the second prenatal visit were excluded. Preterm birth was defined as birth before 37 weeks of gestation, based on the date of the last reported menstrual period. Low birth weight was defined as a birth weight below 2500 grams.

### 2.3. Statistical Analysis

All statistical analyses were performed using SAS (version 9.2, SAS Institute, Cary, NC).

Descriptive analyses included calculation of medians, standard deviations, and frequencies of the exposure, outcomes, and covariates. Categorical proportions were compared using chi-square test and continuous variables were assessed using the Wilcoxon rank-sum test [16].

Using Cox proportional hazards analysis we estimated unadjusted and adjusted hazard ratios (HRs) and 95% confidence intervals (CIs) for the effect of CPT status on time to malaria infection. The initial model contained only CPT exposure and the outcome of interest. Multivariable models were then constructed including covariables known from the literature to be associated with CPT or the outcome of interest including maternal age, CD4 cell count, marital status, education, past medical conditions, rainy season, gravidity, and BAN randomization arm. We examined the proportional hazard assumption graphically using log-log plots and by adding interactions with time to the model. If the assumption was violated, it was relaxed by fitting interactions with categorical or continuous time [17]. We explored rainy season, maternal age, and first pregnancy as modifiers of the association between CPT and the outcome of interest [18]. Covariables found to be important effect measure modifiers were included in the multivariable model through an interaction term with CPT exposure status. To construct the final model, we used a manual, backward elimination, change-in-estimate strategy. Potential confounders were removed from the preliminary full model in order of *P* value magnitude. If the CPT-outcome association changed by less than 10% overall or in any stratum of an interacting variable, a given covariable was not retained [19].

To assess the association between CPT during pregnancy and low birth weight we estimated odds ratios (ORs) and 95% confidence intervals (95% CIs) using multivariable logistic regression. The models were constructed as described above, and effect measure modification was assessed by examining Wald *P* values or the likelihood ratio test for the model with and without the selected interaction term. As with the previous analysis, a manual backward elimination process was used to construct the final model. For analysis of the association between CPT during pregnancy and preterm birth, multivariable log binomial regression was used, due to higher frequency of the outcome (23.6%) [20], following the same procedures for model building as outlined for logistic regression.

The effect of CPT on change in CD4 cell count at 24 weeks postpartum was assessed by constructing separate linear regression models, stratified by whether or not participants were randomized to the maternal antiretroviral regimen. CD4 cell count at screening (conducted at a median time of 14.3 weeks (interquartile range: 9.7, 18.6) before delivery) was included in both linear models. Crude and adjusted CD4 cell counts at 24 weeks were calculated along with 95% confidence intervals. Effect measure modification was assessed by examining the partial *F* test for the model with and without the selected interaction terms [21]. As with the other analyses, a manual backward elimination process was used to develop the final model.

### 2.4. Sensitivity Analysis among Women Ineligible for CPT

Women with a CD4 cell count of 500 cells/*μ*L or greater at screening were ineligible for CPT during pregnancy. We used data from this pseudo “control” group to assess possible unmeasured confounding, that is, whether there were changes in the frequency of incident malaria in the study population between the time periods before and after study implementation of CPT. To do this, we assigned a time-defined exposure which coincided with the rollout of CPT. Women with a CD4 cell count of at least 500 cells/*μ*L who had their second prenatal study visit after 15 August 2006 were considered “exposed” to the later time period. Women with a CD4 cell count of at least 500 cells/*μ*L who delivered before 13 June 2006 were considered “unexposed”. Unadjusted and adjusted HRs for the association between the time-defined exposure and malaria in pregnancy were calculated as described above. In order to compare the frequency of malaria before and after implementation of CPT (i.e., April 2004 to June 2006 versus August 2006 to September 2009), we calculated unadjusted and adjusted HRs using data from women in both the original study population and the “control” population. Three variables were included in this model: exposure to CPT, time period (as previously defined), and CD4 cell count of below 500 cells/*μ*L.

## 3. Results

After excluding 850 women with a CD4 greater than 500 at screening and 197 women with mixed CPT exposure, 468 CPT-unexposed and 768 CPT-exposed mother-infant pairs were eligible for analysis (Table 1). At the screening visit, the median CD4 cell count was lower in CPT-unexposed women (*P* < 0.01).

There were 90 infants of low birth weight with a median birth weight of 2300 grams (interquartile range: 2140, 2400); 33 (36.3%) were born to mothers without CPT exposure and 58 (63.7%) were born to exposed mothers (Table 2). The median birth weight of children born to CPT-unexposed women was 3020 grams and the median birth weight of children born to CPT-exposed women was 3030 grams (*P* = 0.68). The unadjusted OR for the effect of CPT versus SP-IPTp on having a low birth weight infant was 1.08 (95% CI: 0.70, 1.69). None of the covariates explored met the criteria for inclusion in the final model as an effect measure modifier or confounder.

Date of last menstrual period was only available for 624 (50.5%) women, due to a change in data collection procedures. Women without a date of last menstrual period were of lower educational status (*P* = 0.002) and were more likely to deliver a low birth weight infant (*P* = 0.02). Among the women for whom date of last menstrual period was available, 147 delivered preterm; 59 (40.1%) were unexposed to CPT, and 88 (59.9%) were exposed to CPT (Table 2). The median gestational age was 273 days for women unexposed to CPT and 274 days for women exposed to CPT (*P* = 0.89). The RR for the effect of CPT exposure on preterm birth was 1.00 (95% CI: 0.75, 1.34) (Table 2). None of the covariates explored met the criteria for inclusion in the final model as an effect measure modifier or confounder.

**Figure 2:**
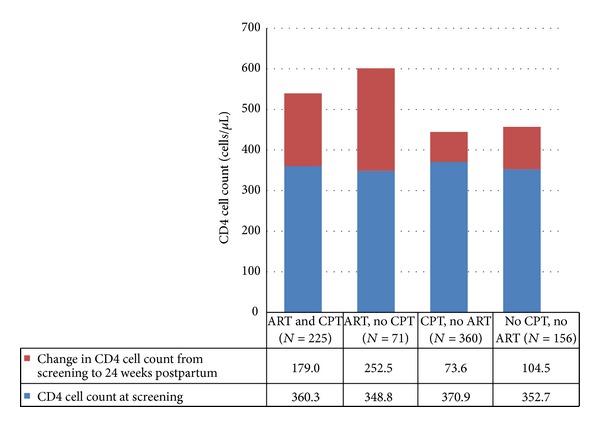
Mean CD4 cell count during pregnancy and mean change in CD4 cell count from screening to 24 weeks postpartum in HIV-infected women during and after pregnancy.

Among the 810 women with CD4 cell count data at prenatal and postnatal study visits, the median time between the two CD4 measurements was 38.4 weeks (interquartile range, 34.0, 42.7). Of these women, 514 did not receive the maternal antiretroviral regimen (156 CPT-unexposed and 358 CPT-exposed) and 296 received the maternal antiretroviral regimen (71 CPT-unexposed and 225 CPT-exposed). Overall, CPT appeared to be associated with lower CD4 cell counts at 24 weeks postpartum (Figure 2). Among women not receiving the antiretroviral regimen, CD4 cell count at 24 weeks postpartum was 33.7 cells/*μ*L (95% CI: 8.8. 58.6) lower among those who received CPT, compared to women who did not receive CPT, after adjustment for CD4 cell count at screening. Similarly, among women who received the antiretroviral regimen, CD4 cell count at 24 weeks postpartum was 77.6 cells/*μ*L (95% CI: 30.1, 125.2) lower among those who received CPT, compared to women who did not receive CPT, adjusted for CD4 cell count at screening. CD4 cell count at screening was the only covariate that met the criteria for inclusion as a confounder or effect modifier in either of the final models.

**Figure 3:**
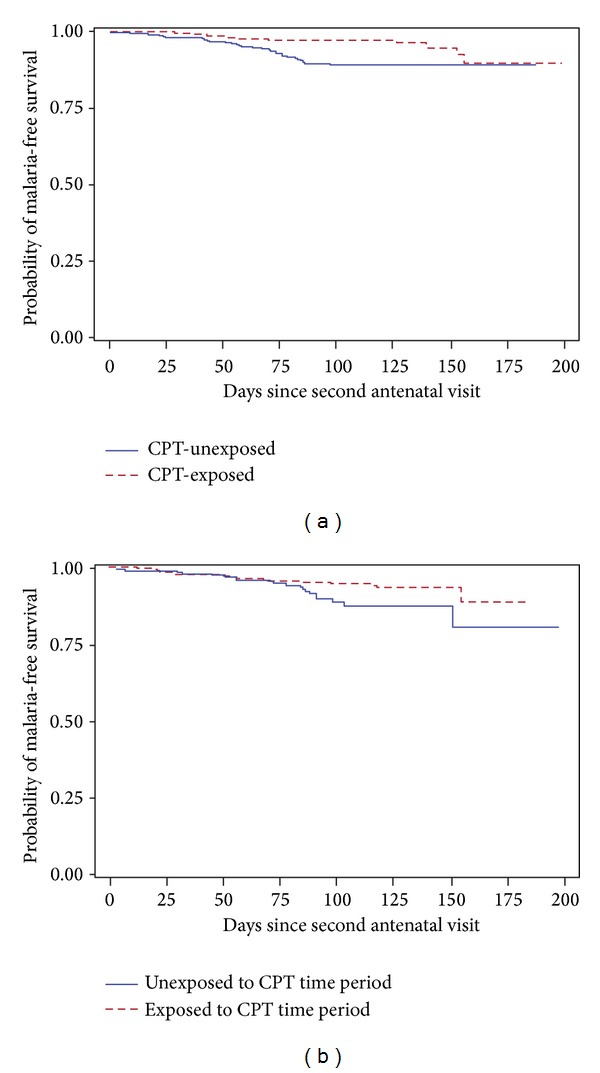
Kaplan-Meier curves illustrating the probability of malaria in HIV-infected pregnant women before and during cotrimoxazole prophylaxis periods for (a) the women with a CD4 less than 500 cells/*μ*L at screening (study population), log rank *P* < 0.0001, and (b) women who had a CD4 of at least 500 cells/*μ*L at screening (“controls”), log rank *P* = 0.0353. All women not receiving CPT received IPTp.

Among the pregnant women included in the analysis, 54 were diagnosed with malaria between the second prenatal study visit (occurring at a mean of 12.7 weeks before delivery) and delivery (Table 2 and Figure 3). Of these 54 women, 33 (61%) were in the CPT-unexposed group and 21 (38.9%) were in the CPT-exposed group. The unadjusted HR for the effect of CPT versus SP-IPTp on incident malaria was 0.35 (95% CI: 0.20, 0.60). None of the covariates explored met the criteria for inclusion in the final model as an effect measure modifier or a confounder.

### 3.1. Sensitivity Analyses among Women Ineligible for CPT

There were 700 pregnant women with a CD4 cell count of at least 500 cells/*μ*L who were included in the sensitivity model to assess the association between study time period and occurrence of malaria. Thirty-eight women were diagnosed with malaria during pregnancy: 55.3% were diagnosed during the earlier time period (between April 2004 and June 2006) and 44.7% were diagnosed during the later time period (between August 2006 and September 2009). The unadjusted HR for the association between malaria and time period of participation was 0.51 (95% CI: 0.27, 0.97) (Figure 3). None of the covariates explored met the criteria for inclusion in the final model, indicating the likelihood of unmeasured confounding.

Next, we included both women with a CD4 cell count of at least 500 cells/*μ*L (the sensitivity analysis population described above) and women with a CD4 cell count of less than 500 cells/*μ*L (our original study population) in order to quantify the effect of CPT on malaria during pregnancy, adjusted for time period of participation. The HR for the association between participation in the later time period and malaria, adjusted for CPT, was 0.52 (95% CI 0.27, 0.98). The HR for the effect of CPT on malaria, adjusted for time period of participation, was 0.66 (95% CI: 0.28, 1.52).

## 4. Discussion

Although there are unique health concerns and considerations for HIV-infected pregnant women, little is known about the effects of CPT in this population. Although our initial analysis indicated that HIV-infected women with CD4 cell counts between 200 and 500 cells/*μ*L treated with CPT during pregnancy had a reduced risk of malaria compared to those who had been treated with SP-IPTp in earlier years, results of our sensitivity analysis suggest this finding resulted at least in part from a reduction in malaria incidence between these two time periods due to unmeasured factors. We found no evidence of protection of CPT against adverse birth outcomes compared to SP-IPTp. In addition, we observed that exposure to CPT may limit the postpartum rebound in CD4 cell count, as observed in this study at 24 weeks postpartum, independent of maternal antiretroviral regimen status.

Our analysis helps to create a more in-depth understanding of the effects of CPT in HIV-infected pregnant women. The WHO CPT recommendations for HIV-infected adults are based on the benefits of prophylaxis including reduced hospitalizations, morbidity, and mortality in HIV-infected patients across varying CD4 levels [1, 3–5, 22–25]. CPT also offers protection against malaria in both HIV-infected and HIV-uninfected adults and children [3, 8, 25, 26]. In our population of pregnant women in Lilongwe, Malawi, CPT started at a median of 12.7 weeks before birth appeared to protect against malaria during pregnancy, compared to SP-IPTp, as has been shown in one other cross-sectional study [10]. However, after consideration of overall trends in malaria incidence during the study, it appears that the observed effect of CPT may be due to an overall decrease in malaria during the later part of the study (and thus may be unrelated to CPT).

In our analysis, CPT was not associated with an effect on preterm birth or low birth weight. Women missing date of last menstrual period were more likely to have a low birth weight infant. However, since there was no difference in distribution of CPT exposure between women with and without the date of last menstrual period, it is unlikely that our results are substantially biased by the missing data. CPT has been associated with a reduced risk of preterm birth in a study among HIV-infected women in Zambia. In that study, women with a CD4 cell count below 200 cells/*μ*L taking CPT had decreased odds for delivery before 34 weeks gestation (OR 0.49, 95% CI 0.24, 0.98) and a trend towards increased birth weight, though this association did not reach significance [7]. The authors of the Zambian study suggest a CPT-related decrease in bacterial and parasitic infections as a mechanism for the reduction in preterm birth, a mechanism which may be less likely to impact birth outcomes in women in our study, who had higher CD4 cell counts.

Regardless of ART and CPT status, there was an increase in median CD4 cell count at 24 weeks postpartum from median prenatal CD4 cell count in our study population. While CD4 cell counts usually decline over time in HIV-infected patients, in pregnant women, CD4 cell count increases in the months postpartum, following a transient decline during pregnancy from hemodilution [27, 28]. Through separate analyses by maternal antiretroviral regimen status, we found that CPT was associated with a lower CD4 cell count at 24 weeks postpartum. The effect of CPT on CD4 cell counts in pregnant women has not been well studied. Results in HIV-infected adults have been mixed. The annual mean rate of decline of CD4 cell count was lower during CPT than before CPT (77 versus 203, *P* < 0.001) in a cohort of HIV-infected patients with a range of CD4 cell counts at baseline in Uganda [2]. In another study of HIV-infected patients in Uganda, CPT was only associated with an effect on CD4 cell count among patients with an initial CD4 cell count of at least 500 cells/*μ*L, in whom CPT was associated with a mean decrease of 22.3 cells/*μ*L (95% CI: 3.7, 42.0) [29].

Although this analysis expands our understanding of CPT in HIV-infected pregnant women, several limitations should be noted. Data on potential confounders which were unmeasured for the analysis of malaria, including use of insecticide-treated nets (ITNs), would enhance our analysis. ITNs were provided to some women in the BAN Study for a period of time beginning in 2007; however, the number provided is not known and there are no data on use of these ITNs by the women included in the analysis. Our ability to assess the effect of time period through inclusion of both study women and “control” women was an important strength of our analysis, allowing us to address confounding that was unmeasured in our primary study population, a major limitation in most observational studies. However, the lack of a true control group should be noted. The “control” population we used to assess temporal changes in malaria had higher CD4 cell counts than our study population, and, therefore, changes in disease incidence in these women may not be a true representation of changes in disease incidence in our study population. While incidence of the other outcomes is more stable, there may also have been unmeasured changes in these which were unrelated to CPT and could confound our results. Of course, we also made multiple assumptions when conducting this sensitivity analysis and thus the findings must be interpreted cautiously. For example, the model assumes the effect of time period is the same regardless of CD4 cell count, that the effect of CPT is the same across time periods, and that the effect of time period is the same across CPT groups. Additionally, pharmacy records were not available to confirm that women received SP-IPTp, the country-wide standard of care for preventing malaria in pregnancy, if they did not receive CPT. We believe that participation in the study and the frequent study visits likely resulted in improved access and adherence to SP-IPTp compared to the national average, but we are unable to determine this without pharmacy records.

We found that CPT in HIV-infected pregnant women with CD4 cell counts between 200 and 500 cells/*μ*L does not have a statistically significant effect on malaria incidence during pregnancy (as compared to SP-IPTp), preterm birth, or low birth weight. CPT may reduce the increase in CD4 cell count seen 24 weeks after birth; however, the duration and any clinical implications of this reduction in CD4 increase were not assessed by this study. Future research may address the effects of CPT in women with CD4 cell counts above 350 cells/*μ*L, as well as in women receiving the more comprehensive antiretroviral treatments now recommended during pregnancy, and could be used to enhance our understanding of the comparative effectiveness of CPT versus SP-IPTp across varying CD4 cell counts and malaria transmission intensities to determine whether forsaking SP-IPTp for CPT is appropriate in all settings.

## 5. Conclusions

Although there are unique health concerns and considerations for HIV-infected pregnant women, little is known about the effects of CPT in this population. Although our initial analysis indicated that HIV-infected women with CD4 cell counts between 200 and 500 cells/*μ*L treated with CPT during pregnancy had a reduced risk of malaria compared to those who had been treated with SP-IPTp in earlier years, results of our sensitivity analysis suggest this finding resulted at least in part from a reduction in malaria incidence between these two time periods due to unmeasured factors. We found no evidence of protection of CPT against adverse birth outcomes. In addition, we observed that exposure to CPT may limit the postpartum rebound in CD4 cell count, as observed in this study at 24 weeks postpartum, independent of maternal antiretroviral regimen status. Additional data about CPT in pregnant women is necessary to enhance our understanding of the effects of CPT beyond its primary effect on opportunistic infections, in order to develop the most beneficial and comprehensive prophylactic treatment for this highly vulnerable population.

## Figures and Tables

**Table 1:** Baseline characteristics of 1236 pregnant women by CPT exposure status.

Characteristic	CPT-unexposed* (*N* = 468)	CPT-exposed* (*N* = 768)	Total population (*N* = 1236)	*P* value^†^
Age (yr)				
Median	25	26	26	0.40
Interquartile range	(22−29)	(23−30)	(23−30)	
CD4 at screening (cells/*µ*L)				
Median	350	362	357	<0.01
Interquartile range	(276−421)	(303−429)	(295−427)	
Maternal education (% >primary)^‡^	38.5	35.3	36.5	0.26
Married (%)	91.7	92.5	92.2	0.62
Mother's first pregnancy (%)	12.4	12.4	12.4	0.99

*Women were considered CPT-unexposed if they gave birth before 13 June 2006; women were considered CPT-exposed if they had their second prenatal visit after 15 August 2006.

^†^ *P* values based on Wilcoxon rank-sum test for continuous variables and chi-square test for binary variables, comparing CPT-exposed and CPT-unexposed groups.

^‡^Level of education was missing for one mother.

**Table 2:** Frequency of outcomes of interest and effect estimates in CPT-exposed and CPT-unexposed pregnant women.

Outcome	CPT-unexposed women*	CPT-exposed women*	Total	Effect estimate^†^ (95% CI)
Malaria during pregnancy	7.2% (33/457)	2.8% (21/751)	4.5% (54/1208)	HR: 0.35 (0.20, 0.60)
Low birth weight^‡^	7.1% (33/467)	7.6% (58/762)	7.4% (91/1229)	OR: 1.08 (0.70, 1.69)
Preterm birth	23.5% (59/251)	23.6% (88/373)	23.6% (147/624)	RR: 1.00 (0.75, 1.34)

Totals for each outcome differ based on available data.

*Women were considered CPT-unexposed if they gave birth before June 13, 2006; women were considered CPT-exposed if they had their second prenatal visit after August 15, 2006.

^†^Effect estimates are unadjusted as no confounders or effect measure modifiers met criteria for inclusion in final models.

^‡^Data were missing for 7 infants.
